# Interface properties of SiO_x_N_y_ layer on Si prepared by atmospheric-pressure plasma oxidation-nitridation

**DOI:** 10.1186/1556-276X-8-201

**Published:** 2013-05-01

**Authors:** Zeteng Zhuo, Yuta Sannomiya, Yuki Kanetani, Takahiro Yamada, Hiromasa Ohmi, Hiroaki Kakiuchi, Kiyoshi Yasutake

**Affiliations:** 1 Department of Precision Science and Technology, Graduate School of Engineering, Osaka University, Suita, Osaka 565-0871, Japan; 2 Research Center for Ultra-Precision Science and Technology, Graduate School of Engineering, Osaka University, Suita, Osaka 565-0871, Japan

**Keywords:** SiO_x_N_y_ film, Interface properties, Interface state density, Atmospheric-pressure plasma, Plasma oxidation-nitridation

## Abstract

SiO_x_N_y_ films with a low nitrogen concentration (< 4%) have been prepared on Si substrates at 400°C by atmospheric-pressure plasma oxidation-nitridation process using O_2_ and N_2_ as gaseous precursors diluted in He. Interface properties of SiO_x_N_y_ films have been investigated by analyzing high-frequency and quasistatic capacitance-voltage characteristics of metal-oxide-semiconductor capacitors. It is found that addition of N into the oxide increases both interface state density (*D*_it_) and positive fixed charge density (*Q*_f_). After forming gas anneal, *D*_it_ decreases largely with decreasing N_2_/O_2_ flow ratio from 1 to 0.01 while the change of *Q*_f_ is insignificant. These results suggest that low N_2_/O_2_ flow ratio is a key parameter to achieve a low *D*_it_ and relatively high *Q*_f_, which is effective for field effect passivation of n-type Si surfaces.

## Background

Silicon oxynitride (SiO_*x*_N_*y*_) is a very useful material for applications in microelectronic and optoelectronic devices due to the possibility of tailoring the film composition and property according to the O/N ratio. Recently, considerable attention has been focused on SiO_*x*_N_*y*_ for anti-reflection coatings and surface passivation films for thin crystalline Si solar cells [1-3]. It has been reported that SiO_*x*_N_*y*_ films with high positive fixed charge density (*Q*_f_) in the range of 10^12^ cm^−2^ is effective for field-effect passivation of n-type Si surfaces [2].

So far, several methods have been applied to grow SiO_*x*_N_*y*_ films. For example, high-temperature (>900°C) processes such as the direct thermal oxynitridation of Si in NO or N_2_O ambient [4,5] and the annealing of SiO_2_ in nitrogen-containing ambient [6,7] have been widely used. However, the high-temperature processes suffer a large thermal budget and a redistribution problem of dopant atoms. Plasma-enhanced chemical vapor deposition (PECVD) process is a low-temperature alternative below 400°C [8-10]. However, the PECVD method needs toxic precursor gases, and it is also noted that the interfacial properties prepared by this method are usually inferior to those of thermal oxides [11], because the deposition method does not consume the substrate Si unlike thermal oxidation. Moreover, in the films prepared by low-temperature PECVD, the concentration of hydrogen atoms in the form of Si-OH and Si-H bonds is high, which are responsible for poor dielectric properties [12]. Nitridation of silicon oxide in low-pressure nitrogen plasma has also been investigated to fabricate SiO_*x*_N_*y*_ at low temperatures [13,14]. In the case of low-pressure nitrogen plasma, the ion bombardment of the film surface is a serious problem to develop highly reliable ultra-large-scale integrated circuits [15]. Recently, we have studied the plasma oxidation of Si wafers to grow SiO_2_ films using atmospheric-pressure (AP) plasma generated by a 150-MHz very-high-frequency (VHF) electric field and demonstrated that high-quality SiO_2_ films can be obtained using He/O_2_ or Ar/O_2_ plasma at 400°C [16,17]. We have also reported that the AP VHF plasma oxidation process at 400°C is capable of producing material quality of SiO_2_ films comparable to those of high-temperature (>1,000°C) thermal oxides. The SiO_2_/Si structure with low interface state density (*D*_it_) around the midgap of 1.4 × 10^10^ cm^−2^ eV^−1^ and moderately high *Q*_f_ of 5.3 × 10^11^ cm^−2^ has been demonstrated [18]. Therefore, addition of N into the SiO_2_ film by AP plasma oxidation-nitridation using O_2_ and N_2_ precursor gas mixture is an alternative approach for obtaining SiO_*x*_N_*y*_ films at a low temperature of 400°C.

The purpose of this work is to present a method for preparing SiO_*x*_N_*y*_ films by AP VHF plasma oxidation-nitridation with a detailed analysis of interface properties of SiO_*x*_N_*y*_ layer by capacitance-voltage (*C-V*) measurements on metal-SiO_*x*_N_*y*_-Si capacitors.

## Methods

**Figure 1:**
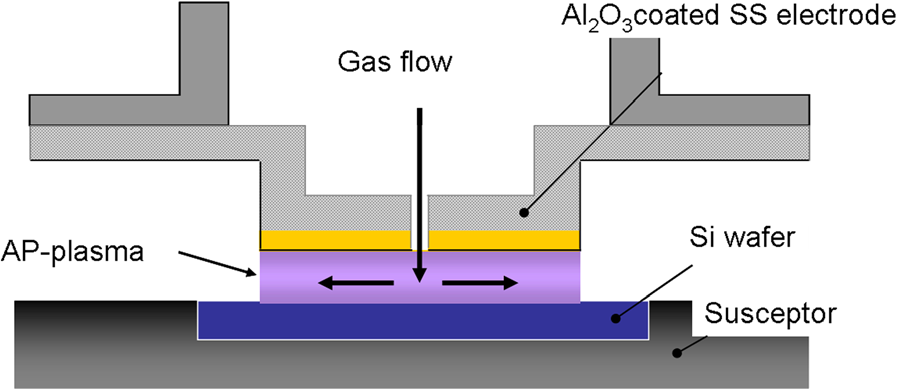
**Schematic illustration of the AP VHF plasma oxidation-nitridation apparatus used in this study.** The electrode is made of stainless steel plate coated with Al_2_O_3_, and its diameter is 50 mm.

The details of the AP VHF plasma apparatus have been reported previously [18]. A schematic illustration of an electrode for AP VHF plasma oxidation-nitridation is shown in Figure 1. In the gap between the substrate and parallel-plate electrode, stable plasma is generated at atmospheric pressure with 150-MHz VHF power using a gas mixture of 1% O_2_/He. N_2_ gas was simultaneously introduced into the AP VHF plasma with gas flow rates of 1, 10, and 100 sccm. The N_2_/O_2_ gas flow ratios were 0.01, 0.1, and 1. The temperature of the Si wafer was fixed at 400°C by monitoring by a thermocouple embedded in the substrate heating stage. The detailed experimental conditions are shown in Table 1.

**Table 1:** Oxidation-nitridation conditions for Si wafer

**Condition**	**Value**
Pressure (Torr)	760
O_2_ concentration (%)	1
He flow rate (slm)	10
O_2_ flow rate (sccm)	100
N_2_ flow rate (sccm)	1,10, and 100
VHF (MHz)	150
VHF power (W)	1,000 to 1,500
Plasma gap (mm)	0.8 to 1
Substrate temperature (°C)	400
Oxidation-nitridation time (min)	9 to 25

The substrates used in the present experiments were n-type (001) CZ-Si wafers (4-in. diameter) with a resistivity of 1 to 10 Ω cm. They were cleaned by a room-temperature chemical cleaning method [19] and were finished by a diluted HF treatment. After AP plasma oxidation-nitridation, some of the samples were subjected to a forming gas anneal (FGA) in 10% H_2_/He for 30 min at 400°C. In order to investigate *Q*_f_ and *D*_it_ of the SiO_*x*_N_*y*_ film, Al/SiO_*x*_N_*y*_/Si metal-oxide-semiconductor (MOS) capacitors were fabricated with 0.5-mm-diameter Al pads by vacuum deposition. A back contacting electrode at the rear Si surface was also made by Al deposition.

The thickness of the SiO_*x*_N_*y*_ layer was determined by ellipsometry (Rudolph Auto EL III) with a wavelength of 632.8 nm. The chemical bonding in the material was investigated by Fourier transform infrared absorption (FTIR) spectrometry (Shimadzu FTIR–8600PC) in the wave number range of 400 to 4,000 cm^−1^. X-ray photoelectron spectroscopy (XPS; ULVAC-PHI Quantum 2000) was used to investigate the depth profile of atomic composition and bonding of atoms in SiO_*x*_N_*y*_ films. High-frequency (HF) and quasistatic (QS) *C-V* measurements were performed using a 1-MHz C meter/CV plotter (HP 4280A) and quasistatic CV meter (Keithley 595), respectively.

## Results and discussion

**Figure 2:**
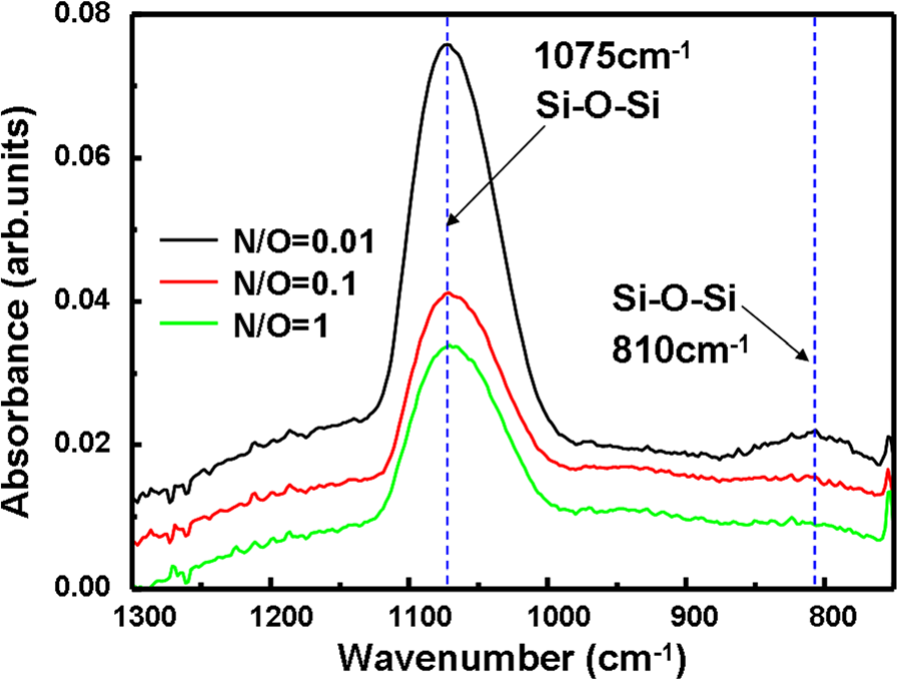
**IR spectra of films prepared by AP VHF plasma oxidation-nitridation under different N**_**2 **_**/O**_**2 **_**flow ratios.**

Thicknesses of films prepared at 400°C for 9 min under N_2_/O_2_ flow ratios of 0.01, 0.1, and 1 were 20.8, 19.5, and 18.9 nm, respectively. (The film thickness was a mean value for measurements of eight different sites on the sample.) Since the difference in the film thickness is small (<±5%), its effect on the interface state properties may be negligible. Figure 2 shows FTIR spectra of the films prepared at 400°C for 9 min under different N_2_/O_2_ flow ratios. The dotted lines in Figure 2 indicate the stretching and bending vibration modes of Si-O-Si bonds at the wave numbers of 1,075 and 810 cm^−1^, respectively. Almost no apparent peak for Si-N stretching mode at 835 cm^−1^ is observed [1], which may be related with the larger dissociation energy of N_2_ than that of O_2_ molecules.

In Figure 2, the strongest peak in IR spectra corresponds to Si-O-Si stretching mode, indicating that the film consists predominantly of SiO_2_. The dielectric constant of the film was calculated using the maximum accumulation capacitance obtained by *C*-*V* curves. The result showed that the dielectric constant was fairly uniform over the sample area with a variation of about 2% and that the average dielectric constants of the films were 4.26 and 4.01 for N_2_/O_2_ flow ratios of 0.01 and 1, respectively. Since the dielectric constants of SiO_2_ and Si_3_N_4_ are 3.9 and 7.5, respectively, nitrogen atoms are considered to be incorporated in the SiO_2_ structure.

**Figure 3:**
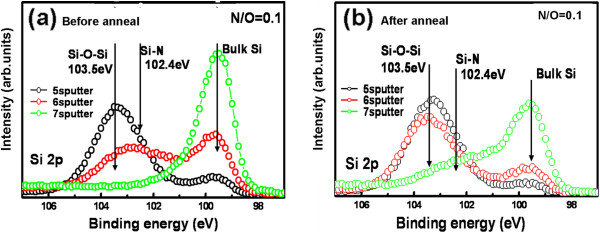
**XPS spectra in Si 2*p *region for SiO**_***x ***_**N**_***y ***_**layer formed by 1% O**_**2 **_**/He AP plasma oxidation-nitridation.** The process is at 400°C for 9 min with a N_2_/O_2_ gas flow ratio of 0.1. (**a**) As-grown sample. (**b**) Annealed sample.

XPS spectra in the Si 2*p* region for the SiO_*x*_N_*y*_ layer formed at 400°C for 9 min with a N_2_/O_2_ gas flow ratio of 0.1 are shown in Figure 3. The Si 2*p* peak observed at 99.7 eV is from the Si substrate and the one at 103.5 eV from Si-O-Si bonding. On the as-grown sample, as shown in Figure 3a, after five times of surface layer sputtering by 10-keV Ar ions (duration of one sputtering is 10 s), Si-O-Si bonding peak is strong, but a small peak from the Si substrate is also seen. By the sixth and seventh sputtering, the Si-O-Si peak decreases and the bulk Si peak increases. It is noteworthy that Si-N bonding at 102.4 eV is also detected. Since the Si-N peak becomes clear before the Si-O-Si peak vanishes, Si-N bonding is supposed to be located at the SiO_2_/Si interface region. In the annealed sample, as shown in Figure 3b, the decrease of the Si-O-Si peak after the sixth sputtering is not significant as compared to that in the as-grown sample and the Si-O-Si peak still remains after the seventh sputtering. The Si-N peak becomes well observable after the seventh sputtering in the annealed sample instead of the sixth sputtering for the as-grown case. However, the tendency of decreasing Si-O-Si peak and increasing bulk Si peak with increasing sputtering time is the same for both as-grown and annealed samples. These results can be understood by considering the increase in SiO_2_ thickness by the annealing and the presence of Si-N bonding at the SiO_2_/Si interface region. The thickness increase in the annealed SiO_2_ sample is considered to be due to the density relaxation of SiO_2_ by the thermal annealing [20,21].

**Figure 4:**
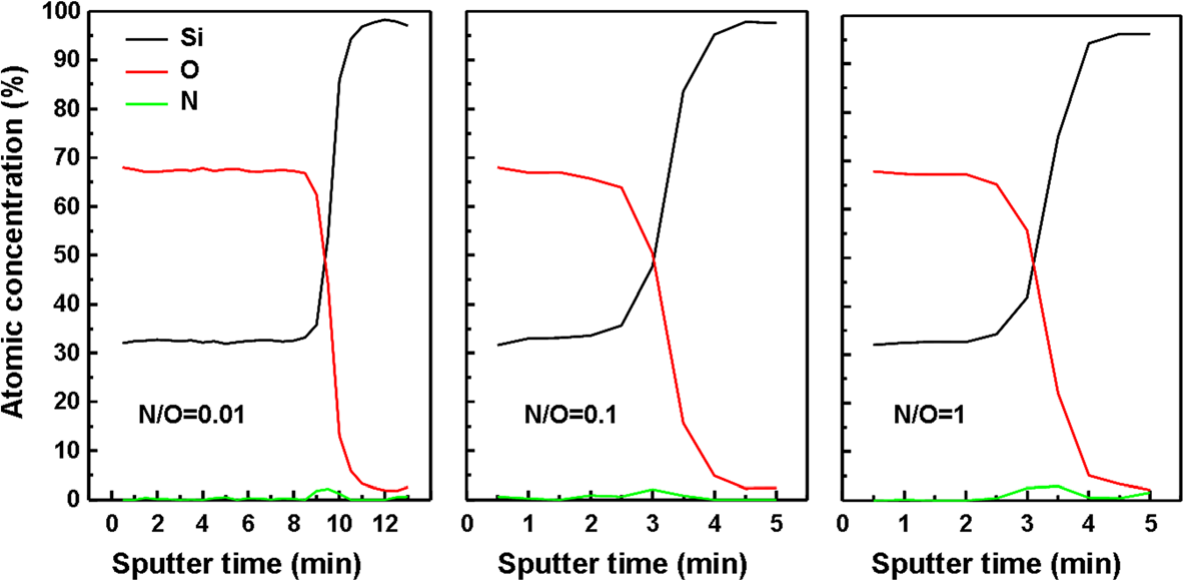
**XPS depth profiles of Si, O, and N concentrations in SiO**_***x ***_**N**_***y ***_**layers.** The layers were prepared by AP VHF plasma oxidation-nitridation process under different N_2_/O_2_ flow ratios.

Figure 4 shows depth profiles of Si, O, and N atom concentrations in SiO_*x*_N_*y*_ films measured by XPS as a function of sputtering time, which reveals that incorporated N atoms (approximately 4%) locate at the film/substrate interface for all the samples. These results are similar to those by the high-temperature process, such as the direct thermal oxynitridation of Si in N_2_O ambient at 1,000°C [5]. According to the thermodynamics of Si-N-O system, nitrogen in bulk SiO_2_ is not thermodynamically stable but may be stable at the interface [22]; therefore, it has been assumed that nitrogen incorporated into the film during oxynitridation (especially in high-temperature N_2_O or NO process) reacts only with Si-Si bonds at or near the interface, not with Si-O bonds in the bulk of the SiO_2_ overlayer. Similarly, we suppose that since the dissociation of nitrogen molecules is not significant in the present case, nitrogen migrates to the Si/SiO_2_ interface during AP plasma oxidation-nitridation.

**Figure 5:**
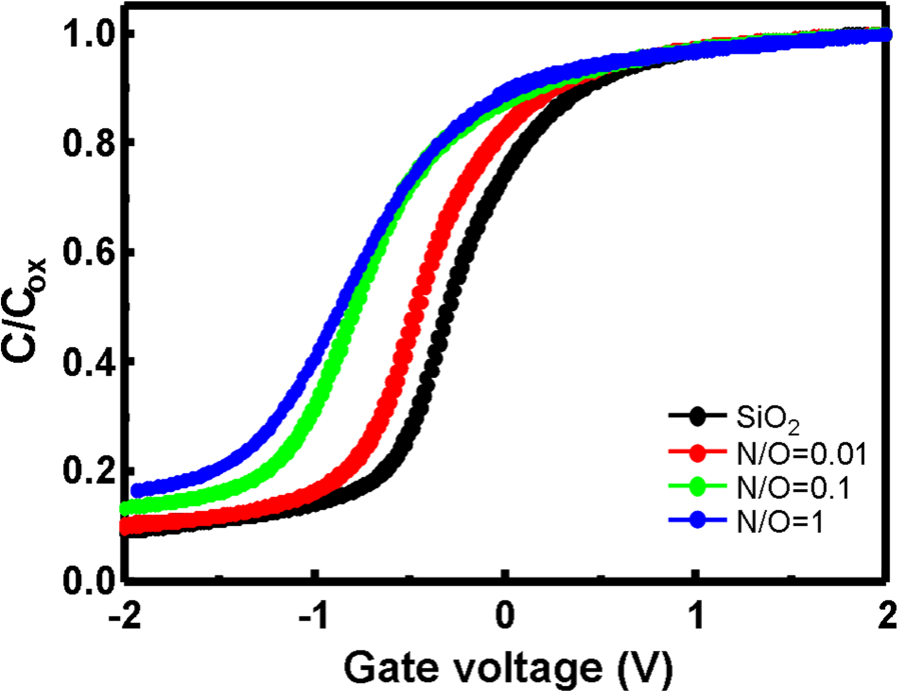
**Typical HF *C *– *V *curves for Al/SiO**_**x **_**N**_**y **_**/Si capacitors utilizing SiO**_**x **_**N**_**y **_**layers prepared by different N**_**2 **_**/O**_**2 **_**flow ratios.** The *C*–*V* curve shifts to a negative gate bias direction with increasing N_2_/O_2_ ratio.

Finally, the interface electrical quality of SiO_*x*_N_*y*_ layers prepared by AP VHF plasma oxidation-nitridation process has been investigated. Figure 5 shows typical HF *C*-*V* curves of the MOS capacitors utilizing SiO_*x*_N_*y*_ layers formed by various N_2_/O_2_ flow ratios. The HF *C*-*V* curve shifts to a negative gate bias direction with increasing N_2_/O_2_ flow ratios, which shows an increase in positive *Q*_f_ with incorporation of more N atoms into the SiO_2_ film (Figure 4). The values of *Q*_f_ have been estimated by flat-band voltage shift to be 5.1× 10^11^, 8.1× 10^11^, and 8.4 × 10^11^ cm^−2^ for N_2_/O_2_ flow ratios of 0.01, 0.1, and 1, respectively.

**Figure 6:**
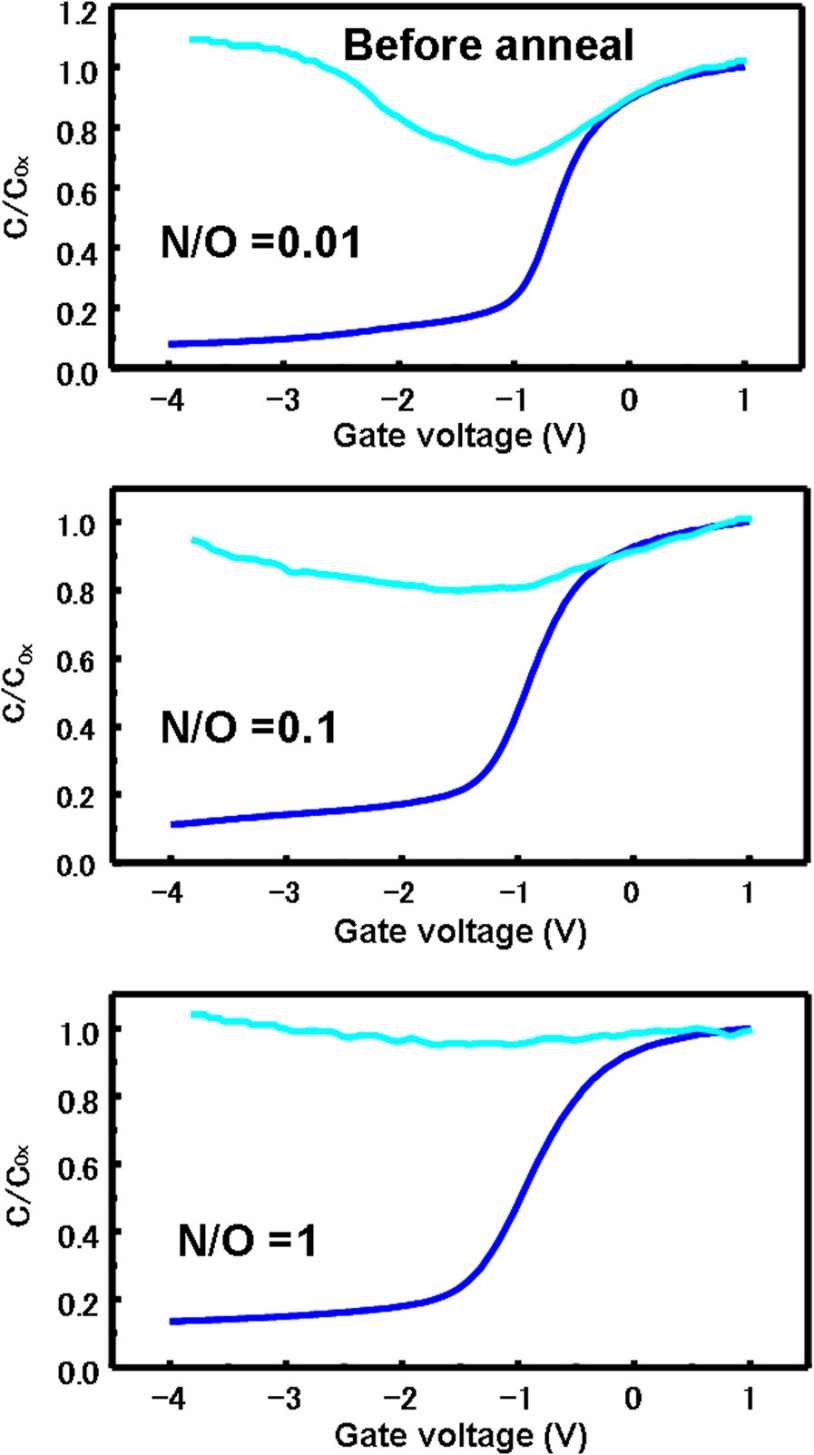
**HF and QS *C*-*V *curves for Al/SiO**_***x ***_**N**_***y ***_**/Si MOS capacitors (before annealing) utilizing SiO**_***x ***_**N**_***y ***_**layers.** The layers were prepared under N_2_/O_2_ gas flow ratios of 0.01, 0.1, and 1.

**Figure 7:**
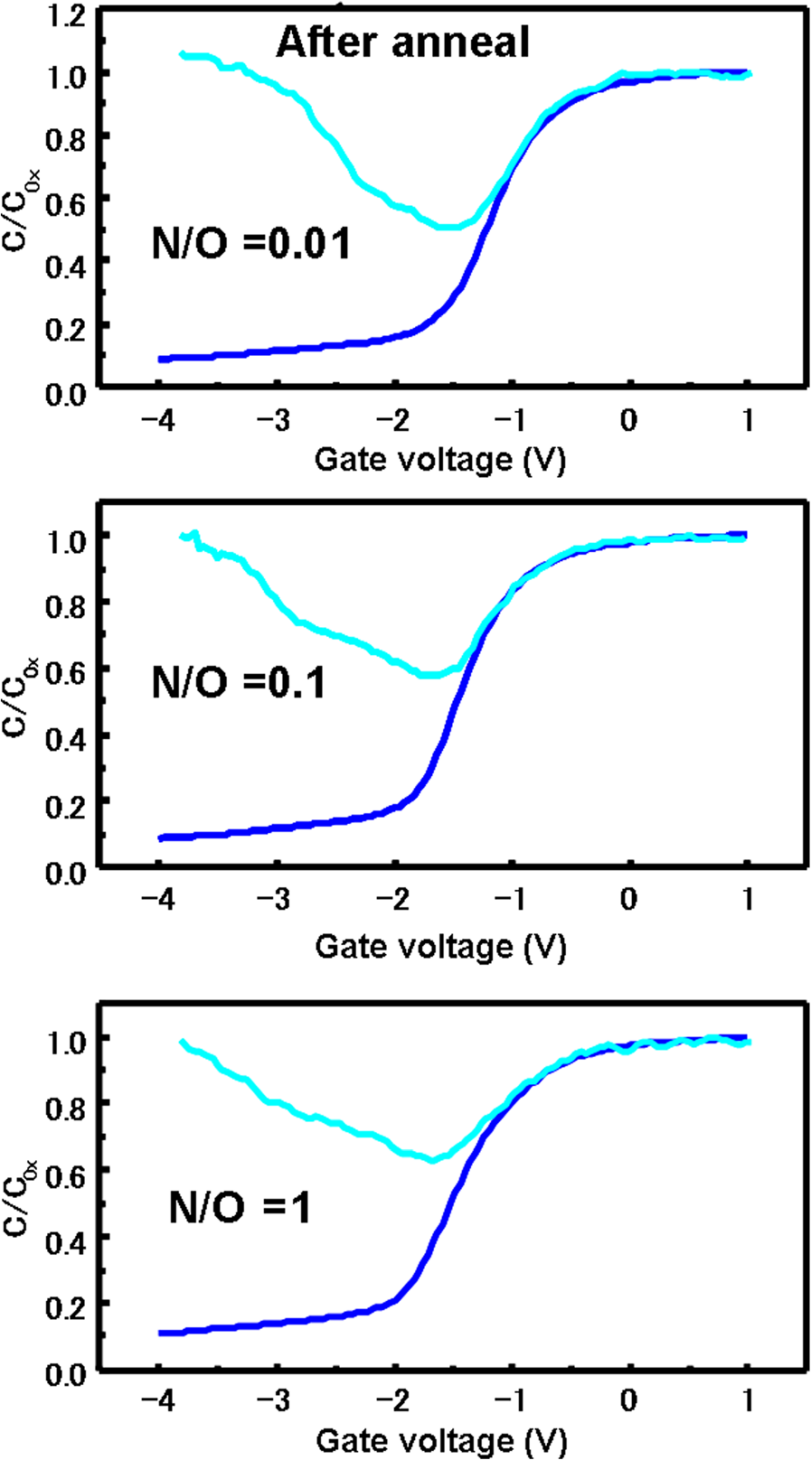
**HF and QS *C*-*V *curves for Al/SiO**_***x ***_**N**_***y ***_**/Si MOS capacitors (after annealing) utilizing SiO**_***x ***_**N**_***y ***_**layers.** The layers were prepared under N_2_/O_2_ gas flow ratios of 0.01, 0.1, and 1.

The HF (blue) and QS (cyan) *C*-*V* curves for Al/SiO_*x*_N_*y*_/Si MOS capacitors before and after FGA are shown in Figures 6 and 7, respectively. The annealed Al/SiO_*x*_N_*y*_/Si MOS capacitors show better interface properties compared with those without FGA. *D*_it_ after FGA were 6.1 × 10^11^, 1.2 × 10^12^, and 2.3 × 10^12^ cm^−2^ eV^−1^ for N_2_/O_2_ flow ratios of 0.01, 0.1, and 1, respectively. It is well known that an introduction of a small amount of nitrogen into the SiO_2_ gate oxide leads to an enhanced defect density in the case of N pileup at the Si/SiO_2_ interface [23]. From our XPS results, when the N_2_/O_2_ gas flow ratio increases, the more N atoms pileup at the Si/SiO_2_ interface during AP plasma oxidation-nitridation; therefore, *D*_it_ increases largely with increasing N_2_/O_2_ flow ratio from 0.01 to 1. The corresponding values of *Q*_f_ were 1.2 × 10^12^, 1.4 × 10^12^, and 1.5 × 10^12^ cm^−2^, respectively. It is noted that *D*_it_ decreases largely with decreasing N_2_/O_2_ flow ratio from 1 to 0.01, while the decrease of *Q*_f_ is insignificant. These results suggest that a significantly low N_2_/O_2_ flow ratio is a key parameter to achieve a small *D*_it_ and relatively large *Q*_f_, which is effective for field-effect passivation of n-type Si surfaces.

## Conclusions

SiO_*x*_N_*y*_ films with a low nitrogen concentration (approximately 4%) have been prepared on n-type (001) Si wafers at 400°C for 9 min by oxidation-nitridation process in AP plasma using O_2_ and N_2_ diluted in He gas. Interface properties of SiO_*x*_N_*y*_ films have been investigated by *C-V* measurements, and it is found that addition of N into the oxide increases both the values of *D*_it_ and *Q*_f_. After FGA, *D*_it_ at midgap decreases from 2.3 × 10^12^ to 6.1 × 10^11^ cm^−2^ eV^−1^ with decreasing N_2_/O_2_ flow ratio from 1 to 0.01, while the decrease of *Q*_f_ is insignificant from 1.5 × 10^12^ to 1.2 × 10^12^ cm^−2^. These results suggest that a low N_2_/O_2_ flow ratio is a key parameter to achieve a low *D*_it_ and relatively high *Q*_f_, which is useful to realize an effective field-effect passivation of n-type Si surfaces.

## Competing interests

The authors declare that they have no competing interests.

## Authors' contributions

ZZ helped in the oxidation-nitridation experiments and sample characterization, and wrote the manuscript. YS and YK performed the atmospheric-pressure plasma oxidation-nitridation of Si wafers and XPS, FTIR, and *C*-*V* measurements. TY, HO, and HK helped in designing the work. KY discussed the results and proofread the manuscript. All authors read and approved the final manuscript.
